# An Updated Review of Tyrosinase Inhibitors

**DOI:** 10.3390/ijms10062440

**Published:** 2009-05-26

**Authors:** Te-Sheng Chang

**Affiliations:** Department of Biological Science and Technology, National University of Tainan, 33 sec. 2 Shu-Lin St., Tainan, Taiwan; E-Mail: mozyme2001@yahoo.com.tw; Tel. +886 6 2606283; Fax: +886 6 2909502

**Keywords:** browning, inhibitors, melanogenesis, tyrosinase

## Abstract

Tyrosinase is a multifunctional, glycosylated, and copper-containing oxidase, which catalyzes the first two steps in mammalian melanogenesis and is responsible for enzymatic browning reactions in damaged fruits during post-harvest handling and processing. Neither hyperpigmentation in human skin nor enzymatic browning in fruits are desirable. These phenomena have encouraged researchers to seek new potent tyrosinase inhibitors for use in foods and cosmetics. This article surveys tyrosinase inhibitors newly discovered from natural and synthetic sources. The inhibitory strength is compared with that of a standard inhibitor, kojic acid, and their inhibitory mechanisms are discussed.

## 1. Introduction

**Figure 1.**
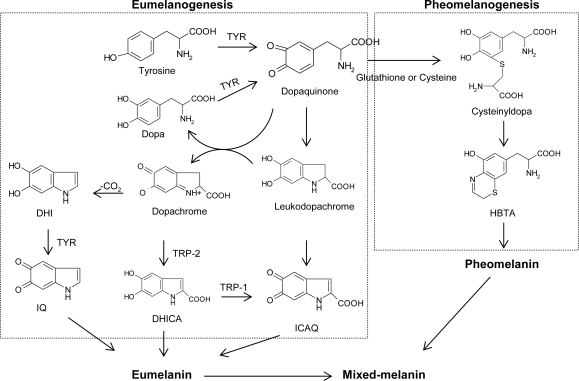
Biosynthetic pathway of melanin [1–4]. TYR, tyrosinase; TRP; tyrosinase related protein; dopa, 3,4-dihydroxyphenylalanine; DHICA, 5,6-dihydroxyindole-2-carboxylic acid; DHI, 5,6-dihydroxyindole; ICAQ, indole-2-carboxylic acid-5,6-quinone; IQ, indole-5,6-quinone; HBTA, 5-hydroxy-1,4-benzothiazinylalanine.

For the past few decades, tyrosinase inhibitors have been a great concern solely due to the key role of tyrosinase in both mammalian melanogenesis and fruit or fungi enzymatic browning. Melanogenesis has been defined as the entire process leading to the formation of dark macromolecular pigments, i.e., melanin. Melanin is formed by a combination of enzymatically catalyzed and chemical reactions. The biosynthetic pathway for melanin formation in various life forms has firstly been elucidated by Raper [1], Mason [2] and recently been modified by Cooksey *et al.* [3] and Schallreuter *et al.* [4] (Figure 1). Melanogenesis is initiated with the first step of tyrosine oxidation to dopaquinone catalyzed by tyrosinase. This first step is the rate-limiting step in melanin synthesis because the remainder of the reaction sequence can proceed spontaneously at a physiological pH value [5]. The subsequent dopaquinone is converted to dopa and dopachrome through auto-oxidation. Dopa is also the substrate of tyrosinase and oxidized to dopaquinone again by the enzyme. Finally, eumelanin are formed through a series of oxidation reactions from dihydroxyindole (DHI) and dihydroxyindole-2-carboxylic acid (DHICA), which are the reaction products from dopachrome. In the presence of cysteine or glutathione, dopaquinone is converted to cysteinyldopa or glutathionyldopa. Subsequently, pheomelanin is formed. In addition to eumelanin and pheomelanin, other “melanin” relying on phenolic monomers different from tyrosine is termed allomelanin. The browning phenomenon in fruit and fungi is also usually related to oxidative polymerization, conceptually similar to melanogenesis. The main difference resides in the fact that allomelanin substantially does not contain dopaquinone-derived motifs as the main monomers in its structure and, on the contrary, is based on other quinoid building blocks. Melanin plays an important role in protecting human skin from the harmful effects of UV radiation from the sun. Melanin also determines our phenotypic appearance.

Although melanin has mainly a photoprotective function in human skin, the accumulation of an abnormal amount of melanin in different specific parts of the skin resulting in more pigmented patches might become an esthetic problem. In addition, enzymatic browning in fruit and fungi is undesirable in, for example, fresh fruits, beverages, vegetables, and mushrooms [6]. Browning after harvest is a common phenomenon in crops such as mushrooms, which decreases the commercial value of the products. Hyperpigmentation in human skin and enzymatic browning in fruits are not desirable. These phenomena have encouraged researchers to seek new potent tyrosinase inhibitors for use in antibrowning of foods and skin whitening. Some tyrosinase inhibitors have been discovered and reviewed before [7–9]; this article surveys tyrosinase inhibitors newly discovered from natural and synthetic sources.

On the other hand, knowledge of melanocyte biology and the processes underlying melanin synthesis has made remarkable progress over the last few years, opening new paths in the pharmacologic approach to the treatment of skin hyperpigmentation. In addition to inhibition of tyrosinase catalytic activity, other approaches to treat hyperpigmentation include inhibition of tyrosinase mRNA transcription, aberration of tyrosinase glycosylation and maturation, acceleration of tyrosinase degradation, interference with melanosome maturation and transfer, inhibition of inflammation-induced melanogenic response, and acceleration of skin turnover. Accordingly, a huge number of depigmenting agents or whitening agents developed by those alternative approaches have been successfully identified and deeply reviewed in many articles [10–16]. Hence, these multidirectional approaches to treat hyperpigmentation are not discussed in this review.

## 2. Biochemical Characteristics and Reaction Mechanism of Tyrosinase

A number of research papers and reviews have already been published on the structural and kinetic aspects of the enzyme tyrosinase [17–22]; therefore, under this section I will briefly discuss tyrosinase’s biochemical characteristics and reaction mechanism.

Tyrosinases (EC 1.14.18.1) catalyze the oxidations of both monophenols (cresolase or monophenolase activity) and *o*-diphenols (catecholase or diphenolase activity) into reactive *o*-quinones. The term tyrosinase refers to its typical substrate, tyrosine. Both tyrosinase activities appear to have broad substrate specificities, although the enzyme has a higher affinity for the L-isomers of the substrates than for the corresponding D-isomers. The first biochemical investigations were carried out in 1895 on the mushroom *Russula nigricans,* whose cut flesh turns red and then black on exposure to air. Since this study, the enzyme has been found widely distributed throughout the phylogenetic scale from bacteria to mammals. The best-characterized tyrosinases are derived from *Streptomyces glausescens*, the fungi *Neurospora crassa* and *Agaricus bisporus*. In fungi and vertebrates, tyrosinase catalyzes the initial step in the formation of the pigment melanin from tyrosine. In plants, the physiological substrates are a variety of phenolics. Tyrosinase oxidizes them in the browning pathway observed when tissues are injured. The enzyme extracted from the champignon mushroom *A. bisporus* is highly homologous with the mammalian ones, and this renders it well suited as a model for studies on melanogenesis. In fact, almost all studies on tyrosinase inhibition conducted so far have used mushroom tyrosinase because the enzyme is commercially available.

**Figure 2.**
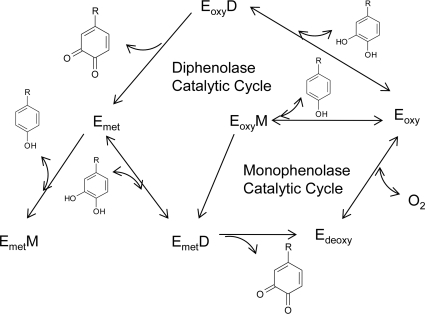
Catalytic cycles of the hydroxylation of monophenol and oxidation of o-diphenol to *o*-quinone by tyrosinase [23–24]. E_oxy_, E_met_, and E_deoxy_ are the three types of tyrosinase, respectively. E_oxy_D, E_oxy_M, and E_met_M are E_oxy_-Diphenol, E_oxy_-Monophenol, and E_met_-Monophenol complexes, respectively.

The notable feature observed in tyrosinases from different sources is that the central copper-binding domain is conserved, which contains strictly conserved amino acid residues, including three histidines [17,23–24]. One tyrosinase molecule can contain two copper atoms, and each atom of the binuclear copper cluster is ligated to three histidines. In the formation of melanin pigments, three types of tyrosinase (oxy-, met-, and deoxytyrosinase, Figure 2) with different binuclear copper structures of the active site are involved. The oxygenated form (oxytyrosinase, E_oxy_) consists of two tetragonal copper (II) atoms, each coordinated by two strong equatorial and one weaker axial N_His_ ligand. The exogenous oxygen molecule is bound as peroxide and bridges the two copper centers. Mettyrosinase (E_met_), similar to the oxy form, contains two tetragonal copper (II) ions coupled through an endogenous bridge, although hydroxide exogenous ligands other than peroxide are bound to the copper site. Deoxytyrosinase (E_deoxy_) contains two copper (I) ions with a co-ordination arrangement similar to that of the met form, but without the hydroxide bridge. The resting form of tyrosinase, i.e., the enzyme as obtained after purification, is found to be a mixture of 85% met and 15% oxy forms.

The above considerations have led to the molecular mechanism for the monophenolase and diphenolase activities of tyrosinase (Figure 2). In the monophenolase cycle, the monophenol can react only with the oxy form and be oxidized to the *o*-quinone, resulting in a deoxy form ready for further dioxygen binding. Oxytyrosinase is, then, regenerated after the binding of molecular oxygen to deoxytyrosinase. If only *o*-diphenol is present (the diphenolase cycle), both the oxy and met forms react with *o*-diphenol, oxidizing it to the *o*-quinone. *o*-Diphenol binds to the oxy form and is oxidized to *o*-quinone, yielding the met form of the enzyme. The latter form transforms another *o*-diphenol molecule into *o*-quinone and is reduced to the bicuprous deoxy form. In most situations, a diphenol is necessary as the reducing agent to obtain the deoxy form, the only one capable of reacting with molecular oxygen and continuing in the catalytic action. For this reason, the monophenolase activity presents a characteristic lag time that exists until a sufficient amount of catechol (needed to reduce the met form to the deoxy one) is produced by the small amount of the oxy form generally present in the resting enzyme preparations. The length of the lag time depends on several factors: the enzyme source; the concentration of monophenol (the lag period being longer when monophenol concentration is increased); the enzyme concentration (with the lag period diminishing, but never totally disappearing, when the enzyme concentration is increased); and finally, the presence of catalytic amounts of *o*-diphenol or transition metal ions, which completely abolish the lag period.

## 3. Tyrosinase Inhibitors

A number of tyrosinase inhibitors from both natural and synthetic sources have been identified. However, the definition of “tyrosinase inhibitor” is sometimes misleading: many authors use that terminology in reference to melanogenesis inhibitors, whose action mainly resides in some interference in melanin formation, regardless of any direct inhibitor/enzyme interaction. Many putative inhibitors are examined in the presence of tyrosine or dopa as the enzyme substrate, and activity is assessed in terms of dopachrome formation. Thus, experimental observation of the inhibition of tyrosinase activity can be accomplished by one of following:

1. Reducing agents causing chemical reduction of dopaquinone such as ascorbic acid, which is used as a melanogenesis inhibitor because of its capacity to reduce back *o*-dopaquinone to dopa, thus avoiding dopachrome and melanin formations.
2. *o*-Dopaquinone scavenger such as most thio-containing compounds, which are well-known melanogenesis inhibitors and react with dopaquinone to form colorless products. The melanogenetic process is therefore slowed until all the scavenger is consumed, and then it goes at its original rate.
3. Alternative enzyme substrates such as some phenolic compounds, whose quinoid reaction products absorb in a spectral range different from that of dopachrome. When these phenolics show a good affinity for the enzyme, dopachrome formation is prevented, and they could be mistakenly classified as inhibitors.
4. Nonspecific enzyme inactivators such as acids or bases, which non-specifically denature the enzyme, thus inhibiting its activity.
5. Specific tyrosinase inactivators such as mechanism-based inhibitors, which are also called suicide substrates. These inhibitors can be catalyzed by tyrosinase and form covalent bond with the enzyme, thus irreversibly inactivating the enzyme during catalytic reaction. They inhibit tyrosinase activity by inducing the enzyme catalyzing “suicide reaction.”
6. Specific tyrosinase inhibitors such as most compounds discussed in this review. The compounds reversibly bind to tyrosinase and reduce its catalytic capacity.

Among the six types of compounds described above, only specific tyrosinase inactivators (5) and inhibitors (6) are regarded as “true inhibitors,” which actually bind to the enzyme and inhibit its activity. General speaking, the mistaken inhibitors exhibit only weak inhibitory activity due to their reactive and consumable properties toward the enzyme or the quinone products. Although some tyrosinase inhibitors exhibited multifunctional activities, the compounds, which are well-known reductive agents, dopaquinone scavengers, or tyrosinase substrates and do not obviously belong to the true inhibitors, are not suitable for comparison with the true inhibitors and are ignored in the present article.

**Scheme 1.**
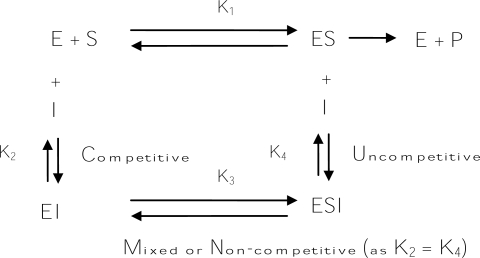
Action mechanism of reversible inhibitors. E, S, I, and P are the enzyme, substrate, inhibitor, and product, respectively; ES is the enzyme-substrate complex, and EI and ESI are the enzyme-inhibitor and enzyme-substrate-inhibitor complexes, respectively.

Usually, “true inhibitors” are classified into four types, including competitive inhibitors, uncompetitive inhibitors, mixed type (competitive/uncompetitive) inhibitors, and non-competitive inhibitors (Scheme 1). A competitive inhibitor is a substance that combines with a free enzyme in a manner that prevents substrate binding. That is, the inhibitor and the substrate are mutually exclusive, often because of true competition for the same site. A competitive inhibitor might be a copper chelator, non-metabolizable analogs, or derivatives of the true substrate. In contrast, an uncompetitive inhibitor can bind only to the enzyme-substrate complex. A mixed (competitive and uncompetitive mixed) type inhibitor can bind not only with a free enzyme but also with the enzyme-substrate complex. For most mixed-type inhibitors, their equilibrium binding constants for the free enzyme and the enzyme-substrate complex, respectively, are different. However, a special case among the mixed inhibitors is the non-competitive inhibitors, which bind to a free enzyme and an enzyme-substrate complex with the same equilibrium constant. In addition to the inhibitory mechanism, inhibitory strength is the primary criterion of an inhibitor. Inhibitor strength is usually expressed as the inhibitory IC_50_ value, which is the concentration of an inhibitor needed to inhibit half of the enzyme activity in the tested condition. However, for the tyrosinase inhibitors in the literature, the IC_50_ values are incomparable due to the varied assay conditions, including different substrate concentrations, varied incubation time, and different batches of commercial tyrosinase. Fortunately, in most studies conducted to discover new tyrosinase inhibitors, a well-known tyrosinase inhibitor such as kojic acid is often used as a positive standard at the same time. Hence, in order to compare the inhibitors described in different literature in a more practical manner, a relative inhibitory activity (RA), which is calculated by dividing the IC_50_ value of kojic acid with that of a newly found inhibitor in the same report, is used to express and compare the inhibitory strength of an inhibitor with others in this review.

**Figure 3.**
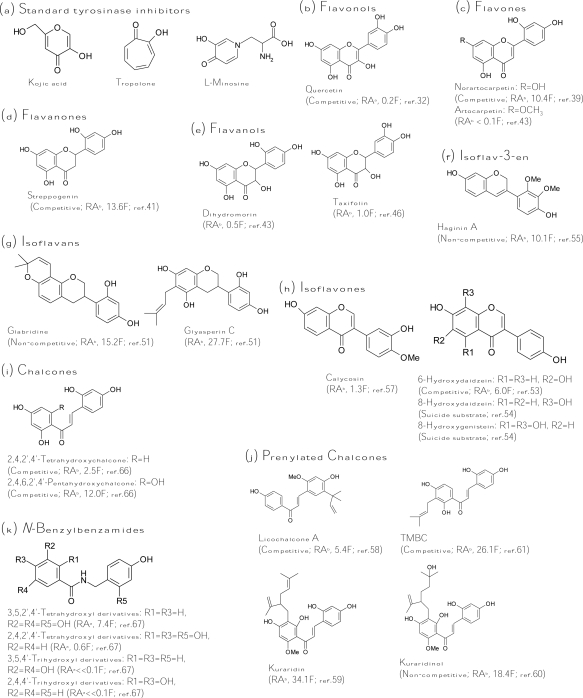
Chemical structures of selected tyrosinase inhibitors belonging to some standard ones (a), flavonoids (b–j) or *N*-benzylbenzamides analogs (k). RA^a^ and RA^b^ are the relative diphenolase and monophenolase inhibitory activity, respectively, against mushroom tyrosinase compared to the standard kojic acid, where 1.0F means one time activity of kojic acid.

Kojic acid (Figure 3a), the most intensively studied inhibitor of tyrosinase, is a fungal metabolite currently used as a cosmetic skin-whitening agent and as a food additive for preventing enzymatic browning [25]. Kojic acid shows a competitive inhibitory effect on monophenolase activity and a mixed inhibitory effect on the diphenolase activity of mushroom tyrosinase. The ability of kojic acid to chelate copper at the active site of the enzyme may well explain the observed competitive inhibitory effect. In addition, kojic acid is reported to be a slow-binding inhibitor of the diphenolase activity of tyrosinase [26]. This means that the active form of tyrosinase, generated in the catalytic cycle in the presence of the substrate, is required before binding of the inhibitor to the enzyme can occur. Other slow-binding inhibitors of tyrosinase are the very potent inhibitor tropolone [27] and the substrate analog l-mimosine [28] (Figure 3a). Strikingly, these slow-binding inhibitors of tyrosinase all contain an α-hydroxyketone group. Kojic acid together with tropolone and l-mimosine are often used as the positive control in the literature for comparing the inhibitory strength of the finding inhibitors.

In addition to the standard tyrosinase inhibitors, a huge number of new inhibitors, especially for those discovered in the last five years, are collected in this review and classified into five major classes, including polyphenols, benzaldehyde and benzoate derivatives, long-chain lipids and steroids, other natural or synthetic inhibitors, and irreversible inactivators based on either the chemical structures or the inhibitory mechanism.

### 3.1. Polyphenols

Polyphenols represent a diverse group of compounds containing multiple phenolic functionalities and are widely distributed in nature. Polyphenols are also the largest groups in tyrosinase inhibitors until now. Since several polyphenols are accepted as substrates by tyrosinase, it depends on the presence and position of additional subsistent whether a polyphenol may act as an inhibitor. Flavonoids are among the most numerous and best-studied polyphenols, that is, benzo-γ-pyrone derivatives consisting of phenolic and pyrene rings. Widely distributed in the leaves, seeds, bark, and flowers of plants, more than 4,000 flavonoids have been identified to date. In plants, these compounds give protection against UV radiation, pathogens, and herbivores [29]. They are also responsible for the characteristic red and blue colors of berries, wines, and certain vegetables. Flavonoids may be subdivided into seven major groups, including flavones, flavonols, flavanones, flavanols, isoflavonoids, chalcones, and catechin. Different classes of flavonoids are distinguished by additional oxygen-heterocyclic rings, by positional differences of the B ring, and by hydroxyl, methyl, isoprenoid, and methoxy groups distributed in different patterns about the rings. The structure of flavonoids is also in principle compatible with the roles of both substrates and (presumably competitive) inhibitors of tyrosinase. Detailed studies have shown that some flavonoids are in fact rather potent inhibitors and discussed in this section. In addition to flavonoids, other polyphenols, which were also identified as tyrosinase inhibitors, contain stilbenes and coumarin derivatives.

#### Flavonols

Many flavonols have been isolated from plants, and some were identified as tyrosinase inhibitors. The inhibitory mode of flavonol inhibitors is usually competitive inhibition for the oxidation of l-dopa by tyrosinase and the 3-hydroxy-4-keto moiety of the flavonol structure acts as the key role in copper chelation [30–31]. In terms of inhibitor strength, the discovered flavonol inhibitors are ranking as follows: quercetin (5,7,3′,4′-tetrahydroxyflavonol) > myricetin (5,7,3′,4′,5′-pentahydroxy-flavonol) > kaempferol (5,7,4′-trihydroxyflavonol) > galangin (5,7-dihydroxyflavonol) >> morin, buddlenoid A, buddlenoid B [32–33]. In addition, the flavonol glycosides of quercetin or kaempferol were found to be less active than their corresponding aglycones [34]. Recently, 6-hydroxykaempferol was synthesized and confirmed to possess two times more activity than kaempferol [35]. Although many flavonols have been identified as tyrosinase inhibitors, all the flavonol inhibitors listed above are very weak inhibitors; the most active flavonol, quercetin (Figure 3b), showed only 20% of the inhibitory strength of kojic acid toward diphenolase activity of mushroom tyrosinase. Hence, it is obvious that these flavonol inhibitors have little potential in applications of skin whitening or food antibrowning.

#### Flavones, flavanones, and flavanols

Citrus peel as a by-product of the citrus juice industry contains a large amount of flavonoids. Some of the flavonoids were identified as tyrosinase inhibitors, including nobiletin (5,6,7,8,3′,4′-hexamethoxyflavone), naringin (5,7,4′-trihydroxyflavanone), and neohesperidin (5,7,3′-trihydroxy-4′-methoxyflavone). However, the inhibitory strength of the three inhibitors was found to be poorly active toward mushroom tyrosinase compared with kojic acid [36–37]. Although no potent tyrosinase inhibitors have been isolated from citrus fruit until now, the ethanolic extract of citrus fruit exhibited *in vitro* inhibitory effects on melanogenesis in melanoma cells and *in vivo* prevention against UVB-induced pigmentation of dorsal skin in brown guinea pigs. The melanogenesis inhibitory activity of citrus crude extracts was found to be mainly attributed to the antioxidant activity of neohesperidin in citrus fruit.

In addition to citrus extracts, the extracts from *Morus* species, which has been well-known as a polyphenol-rich plant and used as a non-toxic natural therapeutic agent, also have high potential in applications as skin-whitening agents due to many potent tyrosinase inhibitors being isolated from different parts of the plant. Mulberroside F (moracin M-6,3′-di-*O*-*β*-glucopyranoside) purified from the leaves of the plant showed the antidiphenolase activity of mushroom tyrosinase to be 4.5-fold higher than that of kojic acid and exhibited an inhibitory effect on melanin formation within melanoma cells [38]. Norartocarpetin (5,7,2′,4′-tetrahydroxyflavone, Figure 3c), isolated from the stem bark of the plant, was found to be 10.4-fold more active than kojic acid against monophenolase activity of mushroom tyrosinase with a competitive inhibition mode (K_I_ = 1.35 μM) [39]. The flavone was also demonstrated to be a slow-binding inhibitor just like kojic acid and tropolone. In addition to the leaves and stem of the plant, the roots of the *Morus* species were also found to contain many very potent tyrosinase inhibitors, including oxyresveratrol [40], norartocarpetin, and streppogenin (5,7,2′,4′-tetrahydroxy-flavavone, Figure 3d) [41]. Oxyresveratrol is a hydroxystilbene with *trans* configuration, and its inhibitory property will be discussed in the later “*silbenes*” section. The chemical structure of streppogenin (a flavanone) is very similar to that of norartocarpetin (a flavone) with the same four substituted hydroxyl groups. It is reasonable that streppogenin possesses an inhibitory property similar to that of norartocarpetin including similarly inhibitory strength on monophenolase activity (IC_50_ = 0.57 and 0.47 μM, respectively) and the same slow-binding and competitive inhibition mode (K_I_ = 0.7 and 0.6 μM, respectively) against mushroom tyrosinase. Moreover, the two potent inhibitors, together with another two analogs, dihydromorin (5,7,2′,4′-tetrahydroxyflavanol, Figure 3e) and artocarpetin (5,2′,4′-trihydroxy-7-methoxyflavone, Figure 3c), were recently isolated from the wood of *Artocarpus heterophyllus* [42–43].

It is very interesting to compare the inhibitory strength toward monophenolase activity of mushroom tyrosinase among the four analogs described above. First, it was found that methoxylation of the hydroxyl group at the C7 position of the flavone skeleton revealed a 100-fold decrease in inhibitory activity by comparing the inhibitory strength between artocarpetin and norartocarpetin. In fact, for most tyrosinase inhibitors, glycosylation and methoxylation of the specific hydroxyl group on the aromatic ring would heavily affect exerting their inhibitory activity. Similar results were also found that forty-five flavonoids glycosides were recently isolated from *Marrubium* species, and all exhibited very low tyrosinase inhibitory activity [44]. The reasons for causing the decreased inhibitory activity by glycosylation and methoxylation of flavonoids include the loss of the specific hydroxyl group, which plays a key role in showing the inhibition and formation of the hydrophilic and stereohindrance effects of the bulky glycoside moiety, which prevent the inhibitor from fitting into the active site of the enzyme. Second, it has been mentioned that norartocarpetin and streppogenin have very similar inhibitory activity and mode toward mushroom tyrosinase. In contrast, dihydromorin, which contains an extra 3-hydroxy group and hence belongs to the flavanol class of flavonoids, exhibited a 20-fold decrease in inhibitory activity compared with that of streppogenin and norartocarpetin. As described in the “*flavonol*” section, it has been demonstrated that the 3-hydroxy-4-keto moiety in the flavonol inhibitors acts as the key role in copper chelation, and loss of the group will completely abolish inhibitory activity [31]. Based on the mechanism, it is difficult to explain the lowered inhibitory capacity of dihydromorin, which contains the 3-hydroxy group and thus forms the 3-hydroxy-4-keto moiety in its structure. Although the 3-hydroxy-4-keto moiety of dihydromorin could form the copper chelating capacity, it seems that the extra 3-hydroxy group in dihydromorin had more drawbacks on tyrosinase inhibition. According to this, the criteria for tyrosinase inhibition by the flavonoids become an interesting issue. Especially, the effects of both the numbers and the positions of hydroxyl groups attached to the flavonoids skeletons on the inhibitory activity of mushroom tyrosinase are the main concern. Kim *et al*. recently systematically tested a large number of flavonoids with different numbers and positions of hydroxyl groups using the fluorescence quenching spectroscopic method [45]. Both the tyrosinase inhibitory activities and copper chelating capacities of the flavonoids were evaluated in the work. From the results, the authors concluded that the enzyme tyrosinase is primarily quenched by the hydroxyl groups of A and B rings on the ether side of the flavonoids (C6 to C8 and C2’ to C4’). According to the finding, the extra 3-hydroxyl group of dihydromorin would indeed reduce its inhibitory activity compared to streppogenin due to the 3-hydroxyl group making dihydromorin more difficult to form a binding complex with the enzyme. Their result could also explain that most flavonols or flavanols inhibitors exhibited only weak to moderate inhibitory strength.

Another flavanol, taxifolin (5,7,3′,4′-tetrahydroxyflavanol, Figure 3e) isolated from the sprout of *Polygonum hydropiper*, showed equal inhibitory activity of kojic acid toward monophenolase activity of mushroom tyrosinase [46]. In an advance study, it is interesting to find that taxifolin would effectively inhibit both the tyrosinase activity of the living cells and cellular melanogenesis as effectively as arbutin, despite its effects on increasing the tyrosinase protein level [47]. In addition to flavonoid monomers, one flavone-flavanone dimmer was isolated from seashore plants, *Garcinia subelliptica*, and revealed to be 3.6-fold more active than kojic acid against the monophenolase activity of mushroom tyrosinase [48].

#### Isoflavonoids

The extracts from the roots and seeds of *Glycyrrhiza* species (Leguminoseae) have long been regarded as an effective constituent for skin-whitening agents in East Asian countries. The melanogenesis inhibitory activity of the extracts mainly comes from the isoflavonoids in the plant. Two isoflavans were purified from the roots of the plant and identified as potent tyrosinase inhibitors. Glabridine (Figure 3g) was the first confirmed inhibitor with 15 times activity of kojic acid and exhibited higher depigmenting activity than that of arbutin [49]. In contrast, glabridine’s analog, glabrene, was found to be 100-fold less active than glabridine [50]. Kinetics study showed that glabridin and glabrene inhibited the enzyme with non-competitive and uncompetitive modes, respectively. Glyasperin C (Figure 3g) was recently isolated from the same part of the plant and shown to be two times more active than glabridin [51]. Although glyasperin C is the most active inhibitor, glabridin had the best melanogenesis inhibitory activity among them.

On the other hand, we isolated several isoflavones derivatives from soybean koji fermented with *Aspergillus oryzae* and demonstrated three hydroxyisoflavones including 6-hydroxydaidzein (6,7,4′-trihydroxyisoflavone), 8-hydroxydaidzein (7,8,4′-trihydroxyisoflavone), and 8-hydroxygenistein (5,7,8,4′-tetrahydroxyisoflavone) as potent tyrosinase inhibitors (Figure 3h) [52–53]. Among them, 6-hydroxydaidzein with 6-fold more than kojic acid acts competitively on the L-tyrosine binding site of the enzyme while the other two 8-hyroxyisoflavones irreversibly inactivate the enzyme and belong to the suicide substrates of tyrosinase (discussed in the “*irreversible inactivators*” section) [54]. It is interesting to note that the position and number of hydroxyl groups in the A ring of the isoflavone structure can strongly affect both the inhibitory strength and the inhibitory mode of the isoflavones to mushroom tyrosinase. For example, an isoflavone with hydroxyl groups at both the C6 and C7 positions in the A ring (6,7,4′-trihydroxyisoflavone) would increase more than 10 times both the inhibitory activity (judged by IC_50_ values) and affinity to the enzyme (judged by Michaelis constants) compared to that of isoflavones either with only one hydroxyl group at the C7 position or without any hydroxyl group in the A ring. Alternatively, the hydroxyl groups at both the C7 and C8 positions (7,8,4′-trihydroxyisoflavone and 5,7,8,4′-tetrahydroxyisoflavone) would completely change the inhibitory mode of the isoflavones from the reversible competitive to the irreversible suicide form. The elucidation of the detailed mechanism of the effects of the hydroxyl groups in the A ring in the isoflavones structure on their inhibitory activity toward tyrosinase needs further study.

In addition to the isoflavonoids from *Glycyrrhiza* plants, Lee *et al.* recently identified other natural isoflavonoids as potent tyrosinase inhibitors. Haginin A (2′,3′-dimethoxy-7,4′-dihdroxyisoflav-3-ene, Figure 3f) isolated from the branch of *Lespedeza cyrtobotrya* was shown to be 10-fold more active than kojic acid against monophenolase activity of mushroom tyrosinase with a non-competitive mode [55]. The compound significantly inhibited melanin synthesis in melanoma cells and decreased UV-induced skin pigmentation in brown guinea pigs. Furthermore, haginin A presented remarkable inhibition on the body pigmentation in the zebrafish model system. Dalbergioidin (5,7,2′,4′-tetrahyroxyisoflavan) was also isolated from L. *cyrtobotrya* and non-competitively inhibited the monophenolase activity of mushroom tyrosinase [56]. Fifty percent of melanin biosynthesis in melanoma cells was inhibited by 27 μM of dalbergioidin, and 80% of cell viability was maintained in this concentration. The third potent inhibitor discovered by the authors is calycosin (4′-methoxy-7,4′-dihydroxyisoflavone, Figure 3h), which showed slighter higher monophenolase inhibitory activity toward mushroom tyrosinase than that of kojic acid. The compound contains two mechanisms to reduce melanogenesis in melanoma cells, including inhibiting tyrosinase activity and reducing the expression of tyrosinase [57].

#### Chalcones

Chalcones consist of two aromatic rings in *trans* configuration, separated by three carbon atoms, of which two are connected by a double bond and the third is a carbonyl group. Some natural prenylated chalcones showed potent tyrosinase inhibitory activity. Three chalcones derivatives, including licuraside, isoliquiritin, and licochalcone A were isolated from the roots of the *Glycyrrhiza* species and competitively inhibited the monophenolase activity of mushroom tyrosinase. Among them, licochalcone A (Figure 3j) was 5.4-fold more active than kojic acid [58]. Another prenylated chalcone, kuraridin (Figure 3j), was isolated from the plant *Sophora flavescens* and identified as a potent tyrosinase inhibitor, which was 34 times activity of kojic acid against monophenolase activity of mushroom tyrosinase [59]. Following, its hydroxyl analog, kuraridinol (Figure 3j), was found to be 18.4-fold more active compared to kojic acid [60]. More interesting, the two prenylated chalcones exhibited significantly more activity than those of their corresponding flavanone analogs. Kuraridin (a chalcone) is 10-fold more active than kurarinone (a flavanone) while kuraridinol (a chalcone) is also 10-fold more active than kurarinol (a flavavone). This emphasizes that tyrosinase inhibitors with a chalcone structure have enhanced potential. Recently, 2,4,2′,4′-tetrahydroxy-3-(3-methyl-2-butenyl)-chalcone (TMBC, Figure 3j), which was isolated from the stems of *Morus nigra*, was proved to be 26-fold more potent than kojic acid in diphenolase inhibitory activity of mushroom tyrosinase [61]. The kinetic study showed that the compound is competitive to the L-dopa binding site of the enzyme with a K_I_ value of 1 to 1.5 μM. Moreover, the melanin content of melanoma cells was reduced to 31% by 30 μM of TMBC treatment. The inhibitory effect of TMBC on melanogenesis was attributed to the direct inhibition of tyrosinase activity, rather than suppression of tyrosinase gene expression.

From the naturally found chalcones listed above, it seems that the 4-resorcinol moiety (2,4-dihydroxyl groups in the aromatic ring) in the chalcone structure is the key substituted group in exerting potent inhibitory activity. Some simple 4-alkylresorcinols were proved to exhibit strong tyrosinase inhibitory activity [62–63]. To evaluate the structure-activity relationship between the numbers and the positions of hydroxyl groups in the chalcone skeleton and the inhibitory activity toward mushroom tyrosinase, the inhibitory activity against mushroom tyrosinase of many chemically synthesized hydroxychalcones was examined. In an early study, Nerya *et al*. reported that the 4-hydroxyl group in the B ring of the chalcone skeleton is the major factor affecting inhibitory potency, because it results in a molecular skeleton closely similar to that of L-tyrosine [64]. Following, they found that 2,4,2′,4′-tetrahydroxychalcone (Figure 3i) possesses the most potent monophenolase inhibitory activity compared with 3,4,2′,4′-tetrahydroxychalcone and 2,4,3′,4′-tetrahydroxychalcone [65]. Therefore, they concluded that 4-resorcinol moiety in the chalcone skeleton plays the key role in exhibiting inhibitory potency. Similarly, Jun *et al.* recently chemically synthesized a series of hydroxychalcones and determined the tyrosinase inhibitory activity [66]. The results were also consistent with that by Nerya *et al*. Moreover, they found that 2,4,2′,4′,6′-pentahydroxychalcone (Figure 3i) is 5-fold stronger than 2,4,2′,4′-tetrahydroxychalcone. The inhibitory mode of the compound is also competitive to the L-tyrosine binding site of the enzyme with a K_I_ value of 3.1 μM. On the other hand, as seen in the description in the earlier “*flavone*” section, it is interesting to note that the identified potent tyrosinase inhibitors with a flavone, flavanone, or flavonol skeleton such as artocarpetin, norartocarpetin, and streppogenin all contain 4-resorcinol in the B ring. Thus, the 4-resorcinol moiety plays an important role in the inhibition of tyrosinase activity not only in chalcones but also in other flavonoid structures.

In similar research with *N*-benzylbenzamide as the structure skeleton, which is analogous to that of chalcone but with an amide moiety (not the alkyl double bond) connecting between the two aromatic rings, the inhibitory activities of *N*-benzylbenzamide derivatives (Figure 3k) were found to rank by 3,5,2′,4′-tetrahydroxyl > 2,4,2′,4′-tetrahydroxyl > 3,5,4′-trihydroxyl > 2,4,4′-trihydroxyl substitutions [67]. Accordingly, it was concluded that the 4-resorcinol in the B ring has more effect than in the A ring on exerting potent inhibitory activity, while the 5-resorcinol in the A ring is preferred to the 4-resorcinol. Very interesting, the structural criterion for exerting potent tyrosinase inhibitory activity in the chalcone-like structure, i.e., the 4-resorcinol moiety in the B ring and the 5-resorcinol in the A ring, is partially consistent with that in the flavone structure studied by the previous fluorescence quenching spectroscopic method, i.e., the hydroxyl groups of A and B rings on the ether side of the flavones (C6 to C8 and C2’ to C4’) [45]. In addition, Khatib *et al*. recently used computer modeling to dock the enzyme crystallographic structure by some 4-resorcinol derivatives with different ester chains and compared the experimental inhibitory IC_50_ values with the calculated free energy and docking energy [68]. The authors’ results emphasize again the importance of the 4-resorcinol skeleton in exerting potent tyrosinase inhibitory activity and the extent to which a lipophilic moiety, combined with the resorcinol skeleton, can contribute to this activity. The additional lipophilic groups affect both the inhibition potency, as well as the ability of the tyrosine to compete with the inhibitors. Such a lipophilic unit, which contains a minor bulky group, was a preferred inhibitor over a long chain or highly bulky functional moiety, as it may interact with the enzyme hydrophobic pocket and augment binding affinity. The conclusion could explain the potent inhibitory activity of prenylated chalcone with 4-resorcinol moiety such as kuraridin and kuraridinol described above.

#### Stilbenes

**Figure 4.**
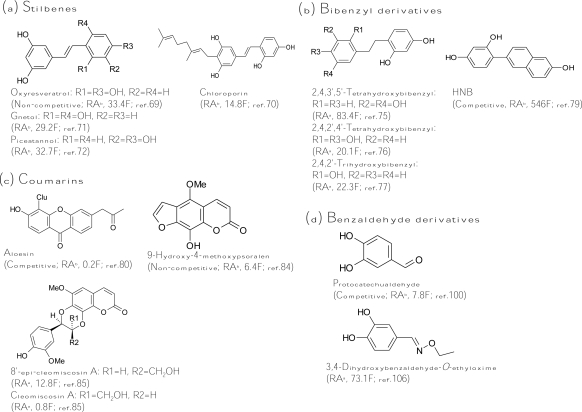
Chemical structures of selected tyrosinase inhibitors belonging to stilbenes (a), bibenzyl derivatives (b), coumarins (c), and benzaldehyde derivatives (d). RA^a^ and RA^b^ have the same meanings as those of Figure 3.

A stilbene consists of an ethene double bond substituted with a benzyl ring on both carbon atoms of the double bond. Oxyresveratrol (2,4,3′,5′-tetrahydroxy-*trans*-stilbene, Figure 4a), which was initially isolated from *Morus alba*, exhibited 32-fold more inhibitory activity than that of kojic acid [40]. The inhibitor acts non-competitively on both monophenolase and diphenolase activity of mushroom tyrosinase. Based on the potent inhibitory capacity, oxyresveratrol reduced pigmentation in melanoma cells. The melanogenesis inhibitory mechanism of the compound was proved to directly inhibit enzyme activity but not affect gene expression [69]. It is worth noting that oxyresveratrol also contains the structure of the 4-resorcinol moiety in the B ring and the 5-resorcinol moiety in the A ring as described in the former “*chalcone*” section. In fact, oxyresveratrol is 50-fold more active than its analog, resveratrol (2,3′,5′-trihydroxy-*trans*-stilbene), which loses the 4-resorcinol moiety in its structure. Taken together with all these findings, the key role of the structural criterion (the 4-resorcinol moiety in the B ring and the 5-resorcinol moiety in the A ring) of an inhibitor in tyrosinase inhibition has been validated in flavones, chalcones, and stilbenes, and this criterion becomes a golden rule for further design of a potent tyrosinase inhibitor.

Following oxyresveratrol, another three hydroxystilbenes were also purified and identified as potent tyrosinase inhibitors. Chloroporin (4-geranyl-3,5,2′,4′-tetrahydroxy-*trans*-stilbene, Figure 4a) was purified from the heartwood of *Chlorophora excelsa* and displayed 14.8-fold inhibitory activity of kojic acid against diphenolase of mushroom tyrosinase [70]. Gnetol (2,6,3′,5′-tetrahydroxy-*trans*-stilbene, Figure 4a), isolated from the roots of *Gnetum gnemon*, exhibited 30-fold more diphenolase inhibitory activity of murine tyrosinase than that of kojic acid [71]. Recently, piceatannol (3,5,3′,4′-tetrahydroxy-*trans*-stilbene, Figure 4a), isolated from grapes and red wine, showed 32.7-fold antimonophenolase activity of kojic acid toward mushroom tyrosinase [72]. The hydroxyl positions of the structure of piceatannol, which contains *o*-(3′,4′-)dihydroxy groups, are significantly different from other naturally found hydroxystilbenes. The *o*-dihydroxy groups play an important role in antioxidant activity (free radical scavenging). In fact, piceatannol has strong suppressive activity in reactive species generation and enhances the reduced/oxidized glutathione ratio in cells. Hence, the inhibitory mechanism of piceatannol on tyrosinase monophenolase activity may be exerted by both direct tyrosinase inhibition and quinone products scavenging, and is not the same with that of oxyresveratrol. However, a detailed kinetic study of the inhibition by piceatannol is lacking.

Aside from naturally occurring hydroxystilbenes, a number of synthetic stilbene derivatives are also good tyrosinase inhibitors. To examine the structure-activity relationship, a variety of hydroxystilbene compounds were synthesized and assayed for their tyrosinase inhibitory activity. The role of the double bond between the two aromatic rings of the stilbene skeleton in the tyrosinase inhibition has been discussed in these studies. By comparing the diphenolase inhibitory activity of murine tyrosinase by *cis*- and *trans*-isomers of 3,3′-dihydroxystilbene, it was concluded that the *trans*-olefin structure of the parent stilbene skeleton is essential for inhibition [73]. Indeed, all the natural hydroxystilbenes possessing potent antityrosinase activity are *trans*-isomers. However, recently, Song *et al.*, concluded the opposite: they found that the diphenolase inhibitory capacity on mushroom tyrosinase by *cis*-3,5-dihydroxystilbene was stronger than that by its corresponding *trans*-isomer [74]. The reasons to explain the obtainted opposite results are not clear. On the other hand, a gnetol analog, dihydrognetol, which loses the double bond between the two aromatic rings, exerted a greatly lowered inhibitory effect on diphenolase activity of murine tyrosinase [73], and it was concluded that the role of the double bond in the stilbene skeleton for tyrosinase inhibition is critical. In contrast, an oxyresveratrol analog, dihydrooxyresveratrol (Figure 4b), which also loses the double bond between the two aromatic rings, showed 8-fold more diphenolase inhibitory activity toward mushroom tyrosinase than its analog, oxyresveratrol [75]. The authors suggested that the higher tyrosinase inhibitory activity of dihydrooxyresveratrol was probably due to its bibenzyl structure, which gave more flexibility and thus allowed the phenolic groups to interact with the enzyme more effectively. Similar results were also found by both Oozeki *et al.* [76] and Vielhaber *et al*. [77]. The former authors synthesized another bibenzyl analog but with 2,4,2′,4′-tetrahydroxyl substitution groups (Figure 4b) and identified the compound to be 20-fold more active than kojic acid while the latter authors found that 4-(1-phenylethyl)1,3-benzenediol (Figure 4b) inhibited mushroom tyrosinase 22 times more effective than kojic acid. These results implied again that the double bond in the stilbene structure is not essential. Despite the undefined inhibitory mechanism of hydroxystilbenes, one Korean research group led by Chung and Suh recently successfully synthesized a new isostere of oxyresveratrol, HNB [4-(6-hydroxy-2-naphthyl)-1,3-bezendiol, Figure 4b], which exhibited 546-fold more inhibitory activity toward monophenolase of mushroom tyrosinase than that of kojic acid [78]. HNB is also the strongest tyrosinase inhibitor published until now. The kinetic study demonstrated that HNB is a competitive inhibitor with a K_I_ value of 4.78 to 6.21 nM. Due to the potent tyrosinase inhibitory activity, it is reasoned that HNB suppressed cellular tyrosinase activity and total melanin content to 27% and 35%, respectively, in melanoma cells at 100 μM concentration, where cells maintained 87% viability [79].

#### Coumarins

Coumarins are lactones of phenylpropanoid acid with an *H*-benzopyranone nucleus. Among the coumarin-type tyrosinase inhibitors, aloesin (Figure 4c) is the famous one, which is a natural hydroxycoumarin glucoside isolated from *Aloe vera*. Aloesin performed more inhibitory activity toward crude murine tyrosinase than mushroom tyrosinase and has been recently used in topically applied cosmetics due to its natural source and multifunctional activity in skin care [80–81]. A coumarin analog, esculetin, was isolated from the seeds of *Euphorbia lathyris* and showed one-quarter of the antityrosinase activity of kojic acid [82]. However, the compound was recently demonstrated to be a substrate of mushroom tyrosinase [83]. Another coumarin analog, 9-hydroxy-4-methoxypsoralen (Figure 4c), was isolated from *Angelica dahurica* and exhibited six times more tyrosinase inhibitory activity than that of kojic acid [84]. Recently, a new coumarin derivative, 8′-epi-cleomiscosin A (Figure 4c), was isolated from the aerial parts of *Rhododendron collettianum* and showed 12.8-fold diphenolase inhibitory activity of kojic acid toward mushroom tyrosinase [85]. Interestingly, another purified compound, cleomiscosin A, had a structure highly similar to that of 8′-epi-cleomiscosin A but exhibited a 14-fold decrease in tyrosinase inhibitory activity compared to that of 8′-epi-cleomiscosin A. The only difference between these two compounds is that the α-proton at the position 8′ for the former, where the β-proton at the same position for the latter. A change in stereochemistry of a single proton that drastically changes the inhibitory activity of the compound is rarely found in the reviewed literature.

### 3.2. Benzaldehyde and Benzoate Derivatives

In the past decade, a large number of benzaldehyde and benzoate derivatives have been isolated from plants and identified as tyrosinase inhibitors, including benzoic acid, benzaldehyde, anisic acid, anisaldehyde, cinnamic acid, and methoxycinnamic acid from the roots of *Pulsatilla cernua* [86], 4-substituted benzaldehydes from cumin [87], 2-hydroxy-4-methoxybenzaldehyde from roots of *Mondia whitei* [88], *p*-coumaric acid from the leaves of *Panax ginseng* [89], hydroxycinnamoyl derivatives from green coffee beans [90], and vanillic acid and its derivatives from black rice bran [91]. The aldehyde group is known to react with biologically important nucleophilic groups such as sulfhydryl, amino, and hydroxyl groups. The tyrosinase inhibitory mechanism of benzaldehyde-type inhibitors comes from their ability to form a Schiff base with a primary amino group in the enzyme [92–93]. In contrast, benzoate inhibits tyrosinase by a copper chelating mechanism and belongs to a typical HA-type acid tyrosinase inhibitor, whose inhibitory mechanism involves the interaction between the non-ionized form of the inhibitor and the copper in the active site of the enzyme [94]. In terms of inhibitory strength, all the naturally occurring benzaldehyde and benzoate derivatives listed above showed only weak-to-moderate tyrosinase inhibitory activity while none are stronger than kojic acid. Recently, flourobenzaldehydes [95], methyl *trans*-cinnamate [96], salicylic acid [97], hydroxybenzaldehydes [98], and 4-β-d-glucopyranosyloxybenzoate [99] were also proved to be weak tyrosinase inhibitors with one to two orders of magnitude of lower antityrosinase activity than that of kojic acid. The most potent natural inhibitor of benzaldehyde type was not identified in plants but in a fungus. Protocatechualdehyde (Figure 4d) was isolated from the fruiting body of *Phellinus linteus* and exhibited 7.8-fold more tyrosinase inhibitory activity than that of kojic acid [100]. It is worth noting that its analog, protocatechuic aldehyde, with two methoxyl groups replacing the two hydroxyl groups showed one order of magnetite of lower activity than that of protocatechualdehyde [101]. Moreover, another analog, protocatechuic acid isolated from black rice bran with a benzoate skeleton, showed another one order of magnetite of lower activity [91]. Thus, it is indicated that both the *o*-dihydroxyl group and the aldehyde group of the protocatechualdehyde structure play important roles in exerting its inhibition activity. On the other hand, to improve the inhibitory strength of the benzaldehyde-type inhibitors, some derivatives were chemically synthesized and assayed to determine their inhibitory activity. 4-Vinylbenzaldehyde [102], 4-alkylbenzaldehyde [103–104], 2-hydroxy-4-isopropyl-benzaldehyde [105], 3,4-dihydroxybenzaldehyde-*O*-ethyloxime (Figure 4d) [106], and 4-butyl-benzaldehyde thiosemicarbazone [107] were successfully synthesized and showed 35-, 100-, 350-, 1500-, and 2000-fold, respectively, more potent activity than that of the precursor benzaldehyde. However, advanced data for cell cytotoxicity and cellular melanogenesis inhibition are lacking for these improved benzaldehyde derivative inhibitors.

Gallic acid (3,4,5-trihydroxybenzoate) has been isolated and identified as a tyrosinase inhibitor from many plants, and its inhibitory mechanism together with those of its ester derivatives has been well studied by Kubo *et al.* [108–110]. They found that gallic acid inhibited diphenolase activity of mushroom tyrosinase with a IC_50_ value of 4500 μM, which is 100-fold lower than that of kojic acid. In addition, gallic acid itself and its short alkyl chain esters (<C10) were oxidized by mushroom tyrosinase as substrates, but the long-chain alkyl chain esters (>C10) inhibited the enzyme without being oxidized. Gallic acid was also found to be very toxic to melanoma cells with cytotoxicity comparable to that of hydroquinone [111]. Recently, Nithitanakool *et al.* isolated both gallic acid and its methyl derivative from seed kernels of *Mangifera indica*, and determined that both compounds were poor inhibitors against diphenolase activity of mushroom tyrosinase with 300- and 30-fold, respectively, lower activity than that of kojic acid [112]. These results were consistent with previous studies. In spite of the poor inhibitory activity of gallic acid itself, some compounds with gallate moiety were found to inhibit mushroom tyrosinase more effectively. Some flavonoids with gallate moiety bonded to the 3-hydroxyl position, including GCG [(+)-gallocatechin-3-*O*-gallate] and EGCG [(−)-epigallocatechin-3-*O*-gallate], were isolated from green tea leaves and showed stronger inhibitory activity [113]. Similarly, three tyrosyl gallates were synthesized and showed stronger inhibitory activity [114]. In recent times, 1,2,3,4,6-pentagalloylglucopyranose isolated from the seed kernels of *M. indica* [112] and the roots of *Paeonia suffruticosa* [115], respectively, was found to be 16-fold more active than gallic acid and inhibited monophenolase activity of mushroom tyrosinase with a non-competitive mode.

### 3.3. Long-chain Lipids and Steroids

**Figure 5.**
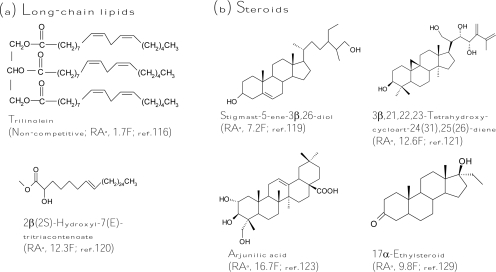
Chemical structures of selected tyrosinase inhibitors belonging to long-chain lipids (a) or steroids (b). RA^a^ and RA^b^ have the same meanings as those of Figure 3.

Recently, several lipids were purified from natural sources and exhibited tyrosinase inhibitory activity. A triacylglycerol, trilinolein (Figure 5a), was isolated from sake lees, which are byproducts of sake production, and proved to be as potent as kojic acid for inhibition of diphenolase activity of mushroom tyrosinase [116]. Based on the findings, including inability of copper chelating, lack of free radical scavenging, and kinetically non-competitive inhibition of the inhibitor, the inhibitory mechanism was proposed by binding of the compound to some site of the tyrosinase, except the catalytic site. A glycosphingolipid, soyacerebroside I, which is composed of a sphingoid base skeleton, an amide aliphatic long-chain fatty acid, and a β-glucopyranose moiety, was isolated from the leaves of *Guioa villosa* and found to inhibit monophenolase and diphenolase activity of mushroom tyrosinase with half-activity of kojic acid [117] while another glycosphinogolipid, cerebroside B, from *Phellinus linteus* showed no inhibitory activity against the enzyme [100]. In addition, *trans* geranic acid from *Cymbopogon citrates* (lemongrass) was recently identified as a tyrosinase inhibitor but with only one-tenth of the inhibitory activity of kojic acid [118].

In addition to long-chain lipids, some steroids were also determined to be tyrosinase inhibitors. Many studies in this field were contributed by the research group of Choudhary and Khan. Three steroids isolated by these authors from the aerial parts of *Trifolium balansae* showed higher diphenolase inhibitory activity toward mushroom tyrosinase than that of kojic acid [119]. Among the steroids, stigmast-5-ene-3β,26-diol (Figure 5b) was 7-fold more active. The authors also found that a long-chain ester, 2β(2*S*)-hydroxyl-7(*E*)-tritriacontenoate (Figure 5a), from *Amberboa ramose* exhibited 12.3-fold more inhibition against the diphenolase activity of mushroom tyrosinase compared to the standard kojic acid [120]. On the other hand, the derivative containing a d-galactopyranosyl moiety at C2 was found to lose one order of magnitude on its tyrosinase inhibitory activity. Like most cases of tyrosinase inhibitors, the presence of the bulky and hydrophilic d-galactopyranosyl moiety interferes with the entrance of the molecule into the active site of the enzyme, thus reducing its inhibitory activity. From the same plant, eight cycloartane triterpenoids were isolated at the same time. A triterpenoid, 3β,21,22,23-tetrahydroxycycloart-24(31),25(26)-diene (Figure 5b), exhibited an extremely potent inhibition (12.6-fold) against diphenolase activity of mushroom tyrosinase when compared with kojic acid [121]. The same research group also purified some triterpenoid glycosides from the roots of *Astragalus taschkendicus*. However, the triterpenoid glycosides showed only inhibitory activity equal to that of kojic acid against mushroom tyrosinase [122]. In advance, Choudhary and Khan purified nine pentacyclic triterpenes from the aerial part of the plant *Rhododendron collettianum*, and all the triterpenes showed potent diphenolase inhibitory activity of mushroom tyrosinase. Among them, arjunilic acid (Figure 5b) was the strongest tyrosinase inhibitor, which was 16.7-fold inhibitory activity of kojic acid [123]. In addition to triterpenoids, the authors also purified many diterpenoids from the aerial parts of *Aconitum leave* [124]. However, most diterpenoids did not inhibit tyrosinase activity. Only one compound, lappaconitine hydrobromide, revealed activity similar to that of kojic acid [125]. Similar to the diterpenoids, few monoterpenoids were found to be strong tyrosinase inhibitors; only a monoterpenoid, crocusatin-K, isolated from the petals of *Crocus sativus*, was proved to display inhibitory activity equal to that of kojic acid [126]. More recently, Wu *et al*. isolated four new sesquiterpenes from the leaves and stems of *Chloranthus henryi* [127] and two new sesquiterpenes dimmers from the leaves of *Chloranthus tianmushanensis* [128], and found that these sesquiterpenes showed either no or weak tyrosinase inhibitory activity. In addition to the original steroids from plants, two 11α-hydroxylated steroid metabolites were isolated from the fungus *Cunninghamella elegans* cultivations feeding with 17α-ethynyl- or 17α-ethylsteroids (Figure 5b) and showed 2.9- and 9.8-fold higher tyrosinase inhibitory activity, respectively, than that of kojic acid [129]. Regarding the difficult permeability of skin, these hydrophobic steroid or long-chain lipid inhibitors seems to have superior potential in the development as skin-whitening agents due to their easier cellular permeability. However, all these lipid inhibitors are lack of cellular or clinical assays for determining their depigmentation activity and remain less utilized by the cosmetics industry until now.

### 3.4. Other Natural and Synthetic Inhibitors

#### Other inhibitors from natural sources

**Figure 6.**
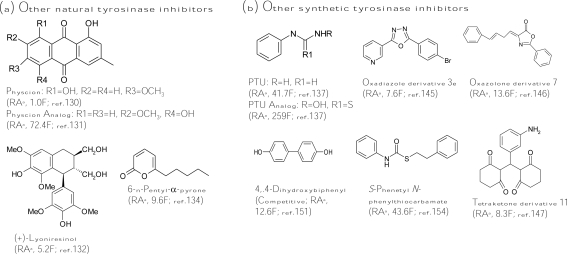
Chemical structures of other tyrosinase inhibitors from natural (a) or synthetic (b) sources. RA^a^ and RA^b^ have the same meanings as those of Figure 3.

Anthraquinones from different plant sources have been widely used since ancient times due to their laxative and cathartic properties. In addition, this class of compounds has shown a wide variety of pharmacological activities, such as antiinflammatory, wound healing, analgesic, antipyretic, antimicrobial, and antitumor activities. Recently, an anthraquinone, physcion (1,8-dihydroxy-2-methoxy-3-methylanthraquinone, Figure 6a), was found to show similar tyrosinase inhibitory activity with that of kojic acid [130]. Interestingly, another anthraquinone, 1,5-dihydroxy-7-methoxy-3-methylanthraquinone (Figure 6a), with hydroxyl and methoxyl groups at different positions compared with those of physcion exhibited a 72-fold increase on antityrosinase activity [131]. Therefore, it is worthy to systematically evaluate the structure-activity relationship between the functional groups attached to the anthraquinone skeleton and the antityrosinase activity. In addition, many lignans isolated from the roots of *Vitex negundo* showed higher tyrosinase inhibitory activity than kojic acid, while the most active lignan from the plant was (+)-lyoniresinol (Figure 6a), whose activity was 5.2-fold higher than that of kojic acid [132]. In addition to the inhibitors from plants, marine beings inhabit virtually any environment in the sea, and they have been shown to produce novel substances with utilities in fine chemicals, drug, and cosmetics products, including tyrosinase inhibitors. One phloroglucinol derivative, dieckol, was isolated from a marine brown alga, *Ecklonia stolonifera*, and displayed three times more activity than that of kojic acid [133]. In addition, a marine-derived fungus *Myrothecium sp*. was found to contain 6-*n*-pentyl-α-pyrone (Figure 6a), which was a potent tyrosinase inhibitor with 9.6-fold inhibitory activity of kojic acid [134]. Recently, another tyrosinase inhibitor was purified from a marine bacteria, *Trichoderma viride* strain H1-7, and showed competitive inhibition toward monophenolase activity of mushroom tyrosinase through binding to a copper active site of the enzyme [135].

#### Other inhibitors from synthetic sources

For smaller molecules, it is easier to rationally design the structures of the molecules by a chemically synthetic method. Improving the tyrosinase inhibitory activity of a known inhibitor by properly changing the substitute groups on its structure is expected. *N*-Phenylthiourea (PTU, Figure 6b) is a well-known inhibitor for inhibiting diphenolase enzyme that belongs to the type-3 copper protein group. The sulfur atom of the compound binds to both copper ions in the active site of the enzyme and blocks enzyme activity [136]. Criton and Le Mellay-Hamon synthesized a series of analogs of *N*-phenylthiourea and put into assays of the inhibitory activity of the analogs against diphenolase activity of mushroom tyrosinase. They found that when the amino group and the sulfur moieties of *N*-phenylthiourea were replaced by *N*-hydroxylamine and oxygen, respectively, the resulting compound (Figure 6b) exhibited six times more activity than that of *N*-phenylthiourea [137]. In another study, the same authors synthesized *N*-(phenylalkyl)cinnamides derived from the coupling cinnamic acid with phenylalkylamines and found that two synthetic compounds showed higher inhibitory activity than that of kojic acid [138]. Similarly, Kang *et al*. designed a series of compounds by combining the structures of two putative tyrosinase inhibitors, kojic acid and caffeic acid, to form new inhibitors, which showed antidiphenolase activity equal to that of kojic acid toward mushroom tyrosinase but enhanced depigmentation activity in melanoma cells [139]. In addition, Shiino *et al*. synthesized analogs of cupferron, which is a well-known metal chelating agent and inhibits competitively both monophenolase and diphenolase activity of mushroom tyrosinase [140], and found that *N*-substituted-*N*-nitrosohydroxylamines inhibited mushroom tyrosinase by interacting with the copper ions at the active site of the enzyme [141]. As removal of nitroso or hydroxyl moiety completely diminished the inhibitory activity, both functional groups were suggested to be essential for chelating activity. Among the derivatives, the authors found that *N*-hydroxybenzyl-*N*-nitrosohydroxylamines showed the best inhibitory activity, which is comparable with that of kojic acid [142]. In advance, they recently reported that these *N*-substituted-*N*-nitrosohydroxylamines inhibited mushroom tyrosinase in a pH-dependent manner due to the non-ionized, electrically neutral form of the compounds [143]. Another research group led by Khan also did a lot of successful work for developing new tyrosinase inhibitors by chemically synthetic methods. The discovered new types of inhibitors included sildenafil [144], oxadiazole [145], oxazolones [146], and tetraketones types [147] (Figure 6b). The strongest inhibitor of each type list above was between 7- and 14-fold stronger than kojic acid. On the other hand, Kim *et al*. focused on developing new type inhibitors containing a selenium atom, which is an essential biological trace element and an integral component of several enzymes. The use of selenium as a nutritional supplement has been popularized recently due to its potential roles as an antioxidant and an anticancer agent. The authors demonstrated that some 1,3-selenazol-4-one derivatives [148], selenourea derivatives [149], and selenium-containing carbohydrates [150] possessed inhibitory activity similar to that of kojic acid against diphenolase activity of mushroom tyrosinase. However, these selenium-containing derivatives were applied in melanogenesis inhibition assays in melanoma cells; most compounds showed both cytotoxicity and depigmenting effects and had restrictions in applications to cosmetics or foods.

Some simple phenyl and biphenyl compounds were also synthesized and identified as potent tyrosinase inhibitors. 4,4′-Dihyldroxybiphenyl (Figure 6b) showed 12-fold more monophenolase inhibitory activity of mushroom tyrosinase than that of kojic acid with a competitive inhibition mode [151] while its glucoside derivatives from the fruit of *Pyracantha fortuneana* displayed only low inhibitory activity [152]. In addition to directly inhibiting tyrosinase activity, 4,4′-dihyldroxybiphenyl was also found to suppress several cellular key parameters in the melanogenic pathway by downregulating the cAMP-dependent protein kinase K signaling pathway and decreasing gene expression of microphthalmia transcription factor, which in turn suppressed tyrosinase expression [153]. In addition, *S*-phenyl *N*-phenylthiocarbamate (Figure 6b) [154] and 4-(2′,4′-dihydroxyphenyl)-(*E*)-3-buten-2-one [155] were recently found to be 44-fold and 6-fold, respectively, more active than kojic acid toward diphenolase activity of mushroom tyrosinase. However, although huge numbers of synthetic inhibitors were successful in inhibiting tyrosinase activity, few have been confirmed in melanogenesis inhibiting activity in cells or skin models.

### 3.5. Irreversible Inactivators

**Scheme 2.**
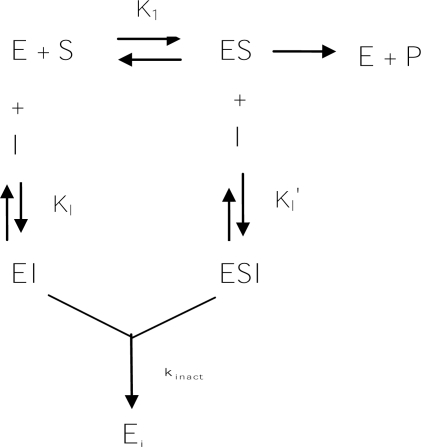
Reaction mechanism of irreversible inhibitors. E and E_i_ are the enzyme and the inactivated enzyme, respectively; S, I, and P are the substrate, inhibitor, and product, respectively; ES, EI and ESI are the intermediates.

In contrast to the huge number of reversible inhibitors has been identified, rarely irreversible inhibitors of tyrosinase were found until now. These irreversible inhibitors, which are also called specific inactivators, can form irreversibly covalent bond with the target enzyme and then inactivate it. However, the tyrosinase irreversible inhibitors are different from non-specifically irreversible enzyme inactivators such as acids or bases, which destroy all protein structures. Instead, they are generally specific for tyrosinase and do not inactivate all proteins; they work by specifically altering the active site of the enzyme. The substrate reaction kinetics method described by Tsou has been widely used in studies of inactivation of various enzymes by different types of inactivation or inhibitions [156]. As shown in Scheme 2, irreversible inhibitors form a reversible non-covalent complex with the enzyme (EI or ESI), and this then reacts to produce the covalently modified “dead-end complex” E_i_. The rate at which E_i_ is formed is called the inactivation rate or *k*_inact_. It is worth noting that irreversible inhibitors display time-dependent inhibition, and their potency therefore cannot be characterized by an IC_50_ value. This is because the amount of active enzyme in a given concentration of irreversible inhibitor will be different depending on how long the inhibitor is pre-incubated with the enzyme.

**Figure 7.**
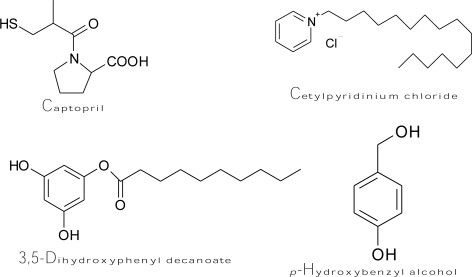
Chemical structures of irreversible tyrosinase inhibitors.

Captopril (Figure 7), an antihypertensive drug [(2*S*)-1-(3-mercapto-2-methylpropionyl)-l-proline], forms both a copper-captopril complex and a disulfide bond between captopril and cysteine-rich domains at the active site of tyrosinase [157]. Accordingly, the drug is able to prevent melanin formation by irreversibly inhibiting monophenolase and diphenolase activity of mushroom tyrosinase in a non-competitive and competitive manner, respectively, as well as by scavenging the generated *o*-quinones to form a colorless conjugate. The kinetic study showed that the value of the rate inactivation constant *k*_inact_ was 1.98 × 10^−4^ μM^−1^s^−1^. H_2_O_2_ is also known as an inactivator of several copper-containing enzymes, such as dopamine β-monooxygenase [158] and mushroom tyrosinase [159]. It was proposed that high concentrations of H_2_O_2_ could oxidize a methionine residue in position 374 of the mushroom tyrosinase active site to methionine sulfoxide, and this event would inactivate the enzyme [160]. Chen *et al.* discovered two other irreversible inhibitors of tyrosinase, including cetylpyridinium chloride [161] and 3,5-dihydroxyphenyl decanoate [162] (Figure 7). The fluorescence spectroscopic results demonstrated that cetylpyridinium chloride can bind to the tyrosinase molecule and induce enzyme conformational changes, which undergo a slow irreversible inactivation of the enzyme. The kinetic study determined the values of the inactivation constants of the inhibitor toward the free enzyme and enzyme-substrate complex to be 3.957 and 6.078 × 10^−3^ s^−1^, respectively. On the other hand, the inhibition mechanism of 3,5-dihydroxyphenyl decanoate was irreversible and competitive/uncompetitive mixed type inhibition with varied inactivation constants ranging from 1.93 to 7.912 × 10^−3^ s^−1^. Recently, *p*-hydroxybenzyl alcohol showed binding capability of mushroom tyrosinase and irreversibly inhibited the enzyme [163]. It was demonstrated that *p*-hydroxybenzyl alcohol (Figure 7) exhibited an inhibitory effect on melanogenesis in melanoma cells at non-cytotoxic concentrations. The suppression of melanin synthesis by the compound was attributed to its behavior in directly inhibiting cellular tyrosinase activity without effects on the expression level of tyrosinase mRNA. In another study, it is very interesting to find that hen egg white lysozyme (HEWL) inhibited mushroom tyrosinase with a reversibly and irreversibly mixed inhibition mechanism [164]. The authors suggested that HEWL binds to glycoside linkage in tyrosinase and induces its conformational change. Some of the bound HEWL continues to non-specifically cleavage glycosidic linkages and induces irreversible inhibition while the other portion of the HEWL-tyrosinase complex restored the diphenolase activity of tyrosinase after depletion of HEWL.

**Scheme 3.**
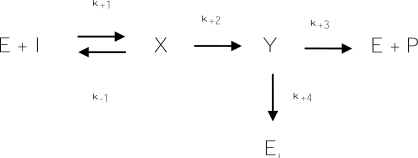
Action mechanism for suicide substrate. E and E_i_ are the enzyme and inactivated enzyme, respectively; P is the product; X is the first intermediate, and Y is another intermediate.

Among the irreversible inhibitors, suicide substrates belong to a special class. It is known that tyrosinase could be irreversibly inhibited by its *o*-diphenol substrates, such as l-dopa and catechol [165]. These substrates were also named suicide substrates or mechanism-based inhibitors. The mechanism of the suicide substrate has been extensively studied by Waley [166], who proposed a simple branched reaction pathway as Scheme 3, in which an intermediate Y may give either an active enzyme and product, or an inactive enzyme. In the scheme, the intermediate Y has a choice of reaction, governed by the partition ratio r, where r = k_+3_/k_+4_. The r value is called the molar proportion for inactivation, i.e., the number of molecules of inhibitors required to completely inactivate one molecule of the enzyme, and may be determined by plotting the fractional activity remaining against the ratio of the initial concentration of inhibitor to that of enzyme. The intercept on the abscissa is 1 + r in the plot, when r > 1 [167]. As in general irreversible inhibitors, the inhibitory strength of a suicide substrate is also not determined by an IC_50_ value but expressed by its r value, where a smaller r value of a suicide substrate means fewer inhibitor molecules are needed to inactivate all enzyme activity and being more powerful inhibition.

**Figure 8.**
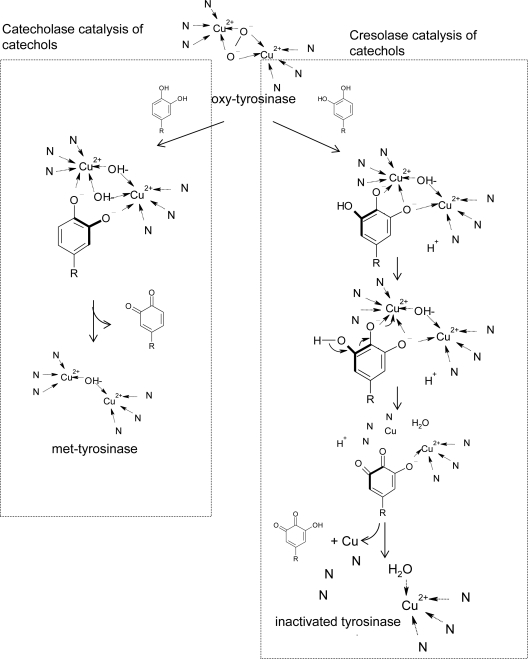
Molecular reaction mechanism of suicide inactivation of tyrosinase by the oxidation of an *o*-diphenol substrate. The curly arrows shows the effect of deprotonation leading to the reduction of copper from bivalent to zero-valent form, elimination of an *o*-quinone and inactivated tyrosinase [168].

In addition to the kinetic reaction mechanism, Land *et al.* recently proposed a model to explain the detailed molecular reaction mechanism of the suicide inactivation of tyrosinase based on the chemical structures of substrates and crystallographic tyrosinase [168]. In their model, the inactivation of tyrosinase during catechol oxidation is due to the catechol being capable of an alternative “cresolase” (monophenolase) presentation (Figure 8). Oxygen addition to the catechol ring by cresolase activity generates an intermediate product that can undergo deprotonation and reductive elimination. This deprotonation leads to the inactivation of the enzyme by formation of a copper atom, which does not bind to the histidine ligands. According to the model, suicide inactivation of tyrosinase depends on anomalous catechol oxidation and the consequence of the formation of zero-valency copper. Their model can explain well the experimental observation that half of the copper is lost from the active site during catechol inactivation. Moreover, the authors in advance used hplc/mass to identify the hydroquinone product in the suicide inactivation of mushroom tyrosinase with 4-methylcatechol as a substrate [169]. Very interestingly, tyrosinase has the following two unique properties: (1) Tyrosinase is able to catalyze oxidations of both monophenols (cresolase activity) and diphenols (catecholase activity) to *o*-quinones, and (2) tyrosinase exhibits suicide inactivation during the oxidation of catechols. In the Land cresolase-dependent model, the two properties are neatly connected, and suicide inactivation is the result of catecholic substrates being processed by the cresolase route.

Although both the kinetic and the molecular reaction mechanism of suicide inactivation of tyrosinase have been well studied, potent suicide substrates have rarely been discovered. Recently, we found that two isoflavones, 7,8,4′-trihydroxyisoflavone and 5,7,8,4′-tetrahydroxyisoflavone (Figure 3h), were potent and unique suicide substrates of mushroom tyrosinase with low partition ratios, low Michaelis constants, and high maximal inactivation rate constants [54]. The partition ratios between molecules of suicide substrate in the formation of the product and in the inactivation of the enzyme were determined to be 81.7 and 35.5, for 7,8,4′-trihydroxyisoflavone and 5,7,8,4′-tetrahydroxy-isoflavone, respectively. In the reviewed literature, 5,7,8,4′-tetrahydroxyisoflavone is the most potent suicide substrate of mushroom tyrosinase until now and has high potential in application as a skin-whitening agent. This compound was in advance creamed in the proper condition [170] and showed higher depigmenting activity than that of ascorbic acid 2-glucoside (AA2G) in an *in vivo* assay conducted with 60 volunteers by our laboratory (unpublished data).

## 4. Conclusions

The commercial availability of mushroom tyrosinase plays a critical role in tyrosinase inhibitor studies, and most research has been conducted with this enzyme, which is well studied and easily purified from the mushroom *A. bisporus*. No matter in terms of inhibitory strength, inhibitory mechanism, chemical structures, or the sources of the inhibitors, the search for new inhibitors based on mushroom tyrosinase has been so successful that various different types of inhibitors have been found in the past 20 years. This success is also based on several fundamental studies in different fields, such as the early finding and the detailed study of tyrosinase as key in melanin biosynthesis, and the deep research into the biochemical, kinetic, reaction mechanistic, and structural aspects of the enzyme.

In spite of this success, it is obvious that much work in this field is still needed. First of all, the commonly used mushroom tyrosinase has some limitations related to the applications of the mushroom tyrosinase inhibitors for human use due to the differences between the fungal tyrosinase and the human one. The mushroom tyrosinase is a cytosol enzyme while the human tyrosinase is membrane bonded [9]. In addition, mushroom tyrosinase is a tetramer in contrast to the monomer type of the human enzyme, which is highly glycosylated during its complex maturation process [21]. However, information regarding the discovered inhibitors to human tyrosinase is very limited; thus, the biochemical information related to kinetic and mechanistic characterizations and to the structure activity relationship between the inhibitors and the human tyrosinase needs further elucidation. To date, no tyrosinase inhibitory study has been performed with human tyrosinase due to the lack of a purified enzyme. Although some studies used a crude extract of human melanocytes as the enzyme source, it is not possible to study the detailed kinetic and mechanistic characterizations of the inhibitors to the specific enzyme in the very complex system. As the primary genetic information of human tyrosinase has been known, utilizing modern genetic engineering to produce recombinant human tyrosinase with suitable properties for easier purification could be possible. Using the purified human tyrosinase to elucidate inhibitory study will aid us considerably in developing the next generation of tyrosinase inhibitors. Moreover, the easier availability of human tyrosinase is also important for the resolution of the X-ray crystallographic structure of the enzyme. Nearly all molecular docking experiments of tyrosinase inhibitors were performed with the recently resolved crystallographic structure of tyrosinase from *Streptomyces glaucescens* [20]; however, the membrane-bonded property of human tyrosinase is expected to show something different in the enzyme structure, which is the prime criterion for inhibitor binding. Thus, the X-ray crystallographic structure of human tyrosinase is also needed and can shed more light on the action mechanism of human tyrosinase and will be helpful in designing more suitable tyrosinase inhibitors for human use.

On the other hand, the use of the inhibitors is primary in the cosmetic industry due to their skin-whitening effects. Since a huge number of tyrosinase inhibitors have been developed, clarifying the validation of these inhibitors in skin-whitening efficiency has become more urgent. Most inhibitors have rarely been incorporated in topically applied cosmetics or cosmeceuticals, often due to a lack of parallel human clinical trials. In addition, most tyrosinase inhibitors listed above are not currently commercially available, especially those from natural sources, and this limits their further evaluation in an *in vivo* study, where usually a large amount is needed for a tested inhibitor. In conclusion, more concrete studies of the found inhibitors with a human clinical point of view are required, and in our experience, this often needs the help and cooperation of cosmetic or biotechnology companies.
